# Effects of Psychotropic Drugs on Ribosomal Genes and Protein Synthesis

**DOI:** 10.3390/ijms23137180

**Published:** 2022-06-28

**Authors:** Zoe S. J. Liu, Trang T. T. Truong, Chiara C. Bortolasci, Briana Spolding, Bruna Panizzutti, Courtney Swinton, Jee Hyun Kim, Srisaiyini Kidnapillai, Mark F. Richardson, Laura Gray, Olivia M. Dean, Sean L. McGee, Michael Berk, Ken Walder

**Affiliations:** 1 The Institute for Mental and Physical Health and Clinical Translation (IMPACT), School of Medicine, Deakin University, Geelong 3220, Australia; zoe.liu@deakin.edu.au (Z.S.J.L.); truongtra@deakin.edu.au (T.T.T.T.); chiara.bortolasci@barwonhealth.org.au (C.C.B.); briana.spolding@deakin.edu.au (B.S.); b.panizzuttiparry@deakin.edu.au (B.P.); courtney.swinton@deakin.edu.au (C.S.); jee.kim@deakin.edu.au (J.H.K.); srisaiyini.kidnapillai@med.lu.se (S.K.); l.gray@deakin.edu.au (L.G.); o.dean@deakin.edu.au (O.M.D.); sean.mcgee@deakin.edu.au (S.L.M.); michael.berk@deakin.edu.au (M.B.); 2 Florey Institute of Neuroscience and Mental Health, Parkville 3010, Australia; 3 Genomics Centre, School of Life and Environmental Sciences, Deakin University, Burwood 3125, Australia; m.richardson@deakin.edu.au

**Keywords:** ribosome, protein translation, psychotropic drug, gene expression, schizophrenia, bipolar disorder, psychiatry, neuroscience, mental disorders

## Abstract

Altered protein synthesis has been implicated in the pathophysiology of several neuropsychiatric disorders, particularly schizophrenia. Ribosomes are the machinery responsible for protein synthesis. However, there remains little information on whether current psychotropic drugs affect ribosomes and contribute to their therapeutic effects. We treated human neuronal-like (NT2-N) cells with amisulpride (10 µM), aripiprazole (0.1 µM), clozapine (10 µM), lamotrigine (50 µM), lithium (2.5 mM), quetiapine (50 µM), risperidone (0.1 µM), valproate (0.5 mM) or vehicle control for 24 h. Transcriptomic and gene set enrichment analysis (GSEA) identified that the ribosomal pathway was altered by these drugs. We found that three of the eight drugs tested significantly decreased ribosomal gene expression, whilst one increased it. Most changes were observed in the components of cytosolic ribosomes and not mitochondrial ribosomes. Protein synthesis assays revealed that aripiprazole, clozapine and lithium all decreased protein synthesis. Several currently prescribed psychotropic drugs seem to impact ribosomal gene expression and protein synthesis. This suggests the possibility of using protein synthesis inhibitors as novel therapeutic agents for neuropsychiatric disorders.

## 1. Introduction

The pathophysiology of schizophrenia (SCZ) remains incompletely understood. An increase in the copy number of ribosomal RNAs has been reported in patients with SCZ [1,2], which is consistent with increased expression of ribosomal genes, a demonstrated characteristic of SCZ [3,4]. Studies using animal models also suggest ribosomal involvement in SCZ. For example, mice with overexpression of SH3 and multiple ankyrin repeat domains 3 (SHANK3), a gene associated with schizophrenia pathogenesis [5,6], exhibited mania-like behaviours [5,7] and enriched ribosome-related genes as identified in The Kyoto Encyclopedia of Genes and Genomes (KEGG) gene library [8]. The ribosome and its associated genes could play a role in the pathological processes of neuropsychiatric disorders, including SCZ.

Ribosomes translate mRNAs into proteins, and their function is an indicator of the integrity of cell morphology and structure. In eukaryotic cells, ribosomes consist of a small (40S) and a large (60S) subunit that assembles over the mRNAs [9]. The small subunit of the ribosome anchors the mRNA so that a set of three nucleotides (a codon) can be presented to a specific tRNA carrying an amino acid at the aminoacyl site (A site). The large subunit of the ribosome links each amino acid to synthesise a polypeptide chain at the peptidyl site (P site) while the empty tRNA is ejected from the ribosome from the exit site (E site) [10]. Mitochondrial dysfunction is implicated in the pathophysiology of multiple psychiatric disorders, including SCZ, bipolar disorder (BD) [11] and major depressive disorder [12], linked to involvement in energy metabolism and redox mechanisms. However, knowledge of mitochondrial ribosomes and their potential role in neuropsychiatric disorders remains scarce. Similar to the cytosolic ribosomes described above, mitochondrial ribosomes are also composed of two subunits (28S and 39S) but reside in the inner mitochondrial membrane rather than the cytosol. They are responsible for translating mitochondrial mRNAs that encode mitochondrial membrane proteins and enzymes for energy production [13].

Multiple studies provide evidence that aberrant protein translation is linked to the pathophysiology of SCZ (reviewed by [14]), possibly associated with reduced synaptic plasticity and hence neurotransmission [15]. BD is associated with stress response in the endoplasmic reticulum, in which some ribosomes are located [16]. Lithium, as a first-choice mood stabiliser for BD, was shown to reverse dysfunction in protein synthesis by inhibiting the phosphorylation of eukaryotic elongation factor-2 (eEF2), an essential regulator of mRNA translation [17]. Moreover, in rodent neurons, the biogenesis and protein composition of ribosomes could be influenced by location and cellular environment, leading to the production of ‘specialised’ ribosomes with exceptional protein translation capacity [18]. This highlights the role of ribosomes in the remote remodelling and repair of neurons along dendrites and axons. This machinery could be impaired in the context of neuropsychiatric disorders.

In line with the above findings, reduced protein translation is implied in SCZ [19] and other psychiatric disorders such as BD [17] and major depressive disorder [20]. At a genetic level, several studies have also reported altered expression of genes involved in the regulation of protein translation. For instance, more pronounced transcriptome alterations in pathways involved in protein synthesis and translation initiation in the dorsolateral prefrontal cortex pyramidal cells were associated with the diagnosis of SCZ in human brain specimens obtained during biopsies [21]. Additionally, functional analyses (including metabolic activity, DNA damage repair and mRNA stability assays) suggested that a microcephalin (*MCPH1*) gene variant with a potential impact on protein translation is associated with the risk of SCZ [22].

Taken together, the ribosome and protein translation may be a target for the development of novel treatments for SCZ and other neuropsychiatric disorders. To test this possibility, we selected some currently prescribed psychotropic drugs with diverse molecular mechanisms of action and investigated the transcriptional effects of each drug on the expression of genes involved in protein translation, including ribosomal genes, in an in vitro model of human neurons. Effects on protein synthesis were also assessed. Given the association between ribosomal dysregulation and SCZ, we hypothesised that psychotropic drugs would alter the expression of ribosomal genes and rates of protein synthesis.

## 2. Results

As revealed by gene set enrichment analysis (GSEA) in the drug-treated neuronal-like post-mitotic (NT2-N) cells, aripiprazole, lithium and risperidone significantly downregulated the expression of KEGG “Ribosome” pathway genes (hsa03010; Table 1) at a transcriptional level. Lamotrigine also showed downregulation, but the effect was no longer significant when the *p*-value was adjusted as a *q*-value to take false discovery rate (FDR) into account. Clozapine increased the expression of genes in this pathway.

To further delineate the transcriptional effects of these four drugs on the ribosome, we narrowed exploration down to the four drugs with significantly adjusted *q*-values and investigated whether there were overall effects of the drugs on the various components of the ribosome.

### 2.1. 40S Subunit

**Figure 1:**
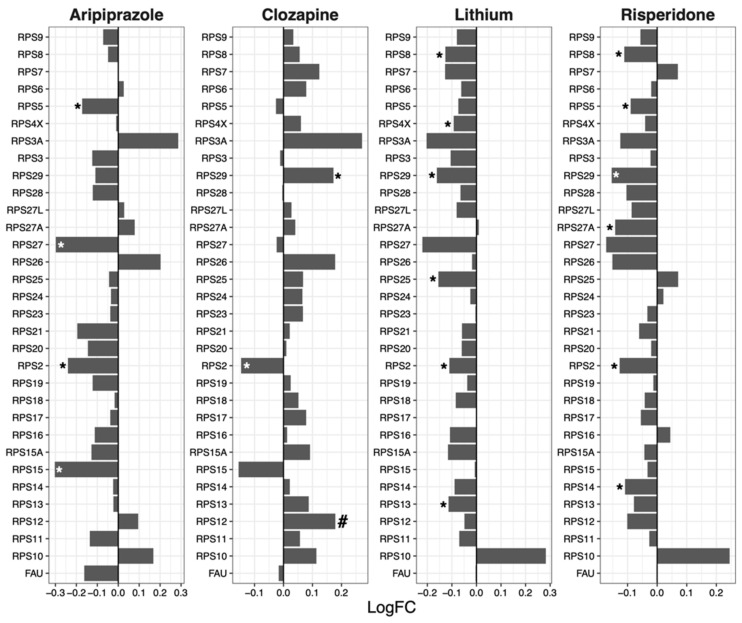
Effects of psychotropic drugs on the expression of genes encoding components of the ribosomal 40S subunit. FC = Fold change, * *p* < 0.05, # *q* < 0.05.

Overall, expression of the genes encoding components of the ribosomal 40S subunit was reduced following treatment of NT2-N cells with aripiprazole (mean log fold change [logFC] = −0.057, *p* = 0.019), lithium (mean logFC = −0.069, *p* = 7.09 × 10^−5^) and risperidone (mean logFC = −0.049, *p* = 0.0021), while expression of these genes was increased by clozapine (mean logFC = 0.050, *p* = 0.0021; Figure 1).

### 2.2. 60S Subunit

**Figure 2:**
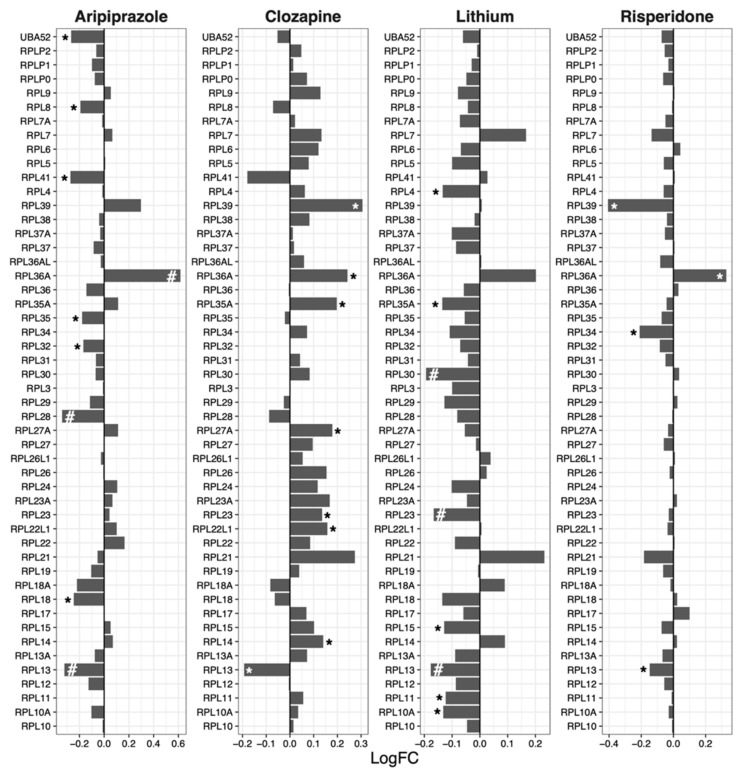
Effects of psychotropic drugs on the expression of genes encoding components of the ribosomal 60S subunit. FC = Fold change, * *p* < 0.05, # *q* < 0.05.

Ribosomal 60S subunit genes were downregulated by aripiprazole (median logFC = −0.027, *p* = 0.010), lithium (median logFC = −0.060, *p* = 2.54 × 10^−7^) and risperidone (median logFC = −0.032, *p* = 5.13 × 10^−5^; Figure 2). Clozapine increased the expression of 60S genes (mean logFC = 0.059, *p* = 0.00011).

### 2.3. Mitochondrial Ribosomal 28S Subunit

**Figure 3:**
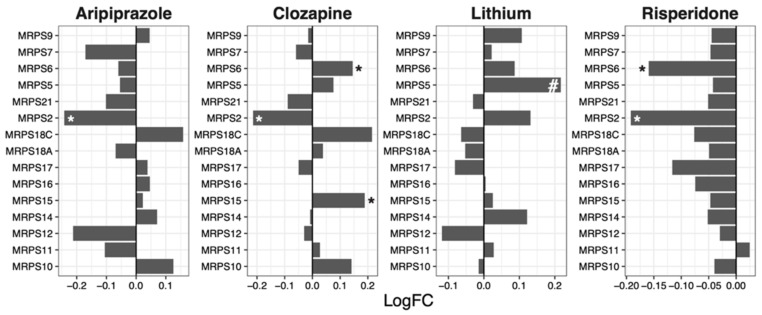
Effects of psychotropic drugs on the expression of genes encoding components of the mitochondrial ribosomal 28S subunit. FC = Fold change, * *p* < 0.05, # *q* < 0.05.

The expression of genes encoding components of the mitochondrial ribosomal 28S subunit was decreased following risperidone treatment in NT2-N cells (median logFC = −0.049, *p* = 1.71 × 10^−5^; Figure 3).

### 2.4. Mitochondrial Ribosomal 39S Subunit

**Figure 4:**
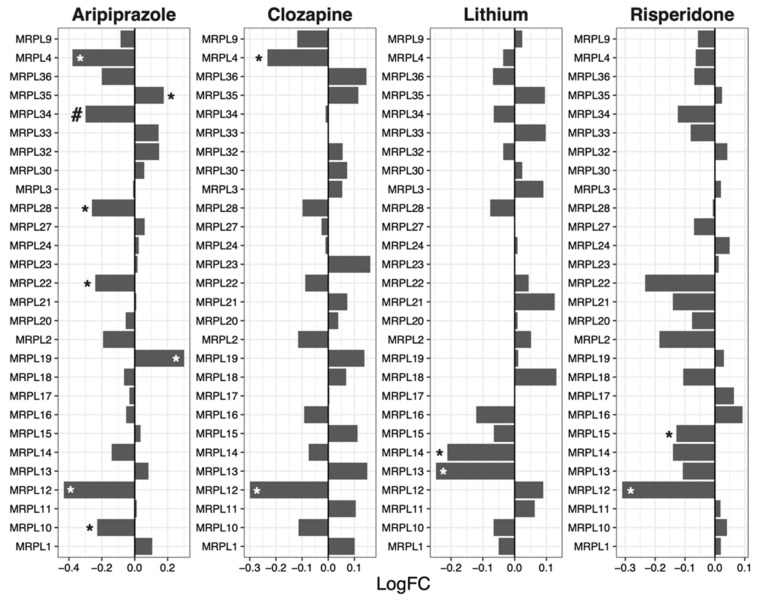
Effects of psychotropic drugs on the expression of genes encoding components of the mitochondrial ribosomal 39S subunit. FC = Fold change, * *p* < 0.05, # *q* < 0.05.

Risperidone (mean logFC = −0.053, *p* = 0.0070) reduced the expression of genes encoding components of the mitochondrial ribosomal 39S subunit (Figure 4).

Table 2 show an overview of the transcriptional regulation of components of the ribosome by the drugs. The expression of genes in the 40S and 60S ribosomal subunits were affected by all four drugs investigated, while the mitochondrial ribosomal subunits were affected by risperidone but not the other three drugs.

### 2.5. Protein Synthesis Assay

**Figure 5:**
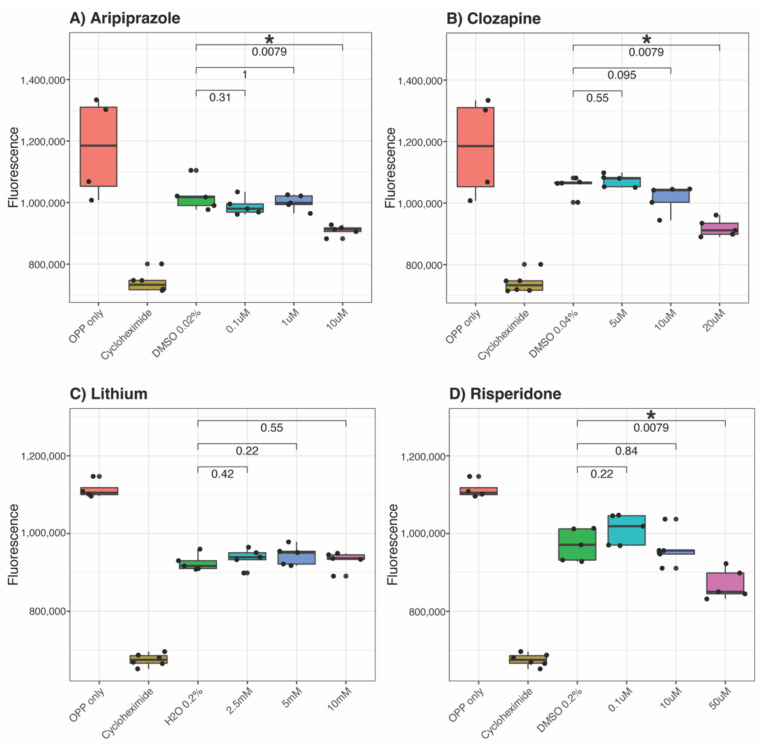
Effects of psychotropic drugs on protein synthesis. NT2-N cells were treated with each of the 4 psychotropic drugs for 24 h: (**A**) aripiprazole, (**B**) clozapine, (**C**) lithium or (**D**) risperidone at 3 different doses. The amount of newly translated proteins generated following drug treatments was compared with a positive control (OPP only), a negative control (cycloheximide) and the corresponding vehicle of the drug (either DMSO or water). Each circle represents a replicate in the experiment. OPP = O-propargyl-puromycin; DMSO = Dimethyl sulfoxide. * represents *p* ≤ 0.01.

We next investigated whether this co-ordinated downregulation of ribosomal genes and elongation initiation factors translated to a measurable difference in protein synthesis. Each drug at three different doses for 24 h was compared to the positive (O-propargyl-puromycin [OPP] only) and negative (cycloheximide) controls and vehicle (either Milli-Q water or dimethyl sulfoxide [DMSO]). We observed that NT2-N cells showed a trend for decreased protein synthesis in response to all four drugs (Figure 5). Aripiprazole, clozapine and risperidone-treated cells showed significantly reduced protein synthesis in a dose-dependent trend compared to the vehicle (Figure 5A,B,D). Lithium did not show significant effects on protein synthesis in the cells (Figure 5C).

## 3. Discussion

In the present study, we utilised NT2-N cells as a model of human neurons, which were treated with eight pharmacologically disparate drugs commonly used in the management of SCZ and BD. To assess the effects of psychotropic drugs on the ribosome and protein synthesis, gene expression profiles were quantified and analysed, and protein synthesis assays were performed.

Differential gene expression and GSEA analyses revealed that four out of eight psychotropic drugs (aripiprazole, clozapine, lithium and risperidone) had significant effects on the expression of ‘Ribosome’ pathway genes (Table 1). More specifically, we identified genes involved in the four key components of the ribosome (40S and 60 subunits and mitochondrial ribosomal 28S and 39S subunits) and quantified the change in their expression following drug treatments. Lithium downregulated the expression of ribosomal genes (Table 1), particularly those involved in ribosomal 40S and 60S subunits (Table 2). Despite this, no significant difference was observed in protein synthesis between cells treated with lithium and vehicle controls (Figure 5C). Lithium has significant transcriptional effects. Consistent with our findings, lithium users (n = 922) in a large case–control study (n = 1450) exhibited reduced expression of genes involved in protein translation machinery compared to lithium-naïve healthy controls [23]. In the post-mortem brains of BD cases who were treated with lithium, the expression of the *RPS23* (ribosomal protein S23) gene was downregulated by 20% compared to healthy controls [24]. Alterations in functional ribosomal genes are linked to tissue-specific defects and developmental disorders [25]. For instance, a mutation in the *RPS23* gene reduced US12 protein stability and its function to correctly decode mRNA, leading to dysmorphic phenotypes in children [26]. Similarly, the loss of the *RPL11* gene (encoding for ribosomal protein UL5, a component of the large subunit of the ribosomal complex) resulted in the disrupted morphology of the developing brain in zebrafish embryos [27]. The above cases reveal the specificity of effects, with lithium treatment decreasing the expression of genes involved in protein synthesis whilst the loss of similar genes has detrimental effects during development. This further highlights the importance of differentiating the developmental roles of genes from the roles played in pathophysiology or treatment response.

Another drug that showed contrasting GSEA and protein synthesis results is clozapine, which upregulated ribosomal genes (Table 1) whilst protein synthesis was downregulated (Figure 5) after drug treatment. Notably, the expression of specific ribosomal genes was significantly reduced by clozapine, in line with the protein synthesis direction. Such genes include *RPS2* (Figure 1), *RPL13* (Figure 2), *MRPS2* (Figure 3), *MRPL4* and *MRPL12* (Figure 4). Multiple potential roles of these ribosomal genes have only been recently explored. These include tumour [28] and bone [29] cell growth, immune responses [30,31,32], metabolic [33,34] and mitochondrial diseases [33,35]. In those studies, the downregulation/dysfunction in genes was associated with the occurrence or increased risk of diseases, reinforcing the need for tight regulation of ribosomal genes for homeostasis. Given clozapine’s unique properties and clinical profile [36], caution is warranted in the interpretation of this result.

Reverse associations between gene expression profiles and protein synthesis are also observed in the literature. When comparing transcriptional data with protein content levels, it is not uncommon to find varying outcomes regardless of the approach to quantifying transcripts and proteins [37,38,39,40]. The link between genomics and proteomics heavily depends on the introduction of a gene-specific RNA-to-protein ratio [41]. This approach, however, has not been widely used because the ratio and calculation methods can vary greatly between cells of different origins and hence requires extensive optimisation. Different experimental models or tissue sources also contribute to mismatches between gene expression and protein data [42]. It is possible that the expression of genes encoding other enzymes capable of modulating protein synthesis was post-transcriptionally affected by clozapine. While more evidence is necessary to assess such a possibility, clozapine was associated with the oxidation of protein products [43] and interacted with an anti-proteinase [44] responsible for the modulation of protein degradation. Taken together, clozapine may affect the activity and susceptibility to proteolyse proteins [45] and, indirectly, the total amount of protein products detected.

Aripiprazole and risperidone reduced the overall expression of ribosomal genes (Table 1) and levels of newly synthesised proteins (Figure 5). Given that an increase in the expression of ribosome-related genes is a feature of SCZ in both humans and animal models [2,3,8], our results suggest that the therapeutic effects of aripiprazole and risperidone may be, at least in part, achieved via the modulation of ribosomal gene expression and reduced protein synthesis. There is little information on aripiprazole’s direct effects on ribosomal activities or protein production. Nevertheless, indirect evidence suggests aripiprazole may downregulate protein synthesis and cell growth. For instance, aripiprazole minimised the symptoms of dysregulated reward processes in mice by dampening the neuroplasticity mechanisms to reduce neuroadaptations [46]. Additionally, aripiprazole inhibits the activity of glycogen synthase kinase-3β (GSK-3β) [47]. GSK-3β promotes the function of p70 ribosomal protein S6 kinase 1 (S6K1), a key modulator of protein synthesis and cell proliferation [48]. Therefore, it is possible that aripiprazole treatment suppressed protein synthesis by hindering GSK-3β and S6K1 activities. On the other hand, risperidone negatively regulates protein synthesis and cell proliferation. Risperidone decreases the level of proteins belonging to the mammalian target of the rapamycin (mTOR) signalling pathway in human cell lines [49]. The mTOR pathway is critical in regulating protein synthesis in the context of neurodevelopment and synaptic plasticity [50,51,52]. Furthermore, risperidone impedes the proliferation and differentiation of pre-osteoblasts, linked to osteoporosis as a side effect after long-term use of the drug [53]. Taken together, the present study suggests a link between aripiprazole/risperidone treatment and the reduction in ribosomal gene expression and protein synthesis.

There are some limitations in the present study. Firstly, the use of the NT2-N cell line may not fully represent neuropsychiatric disorders and their pathophysiological phenotypes. Secondly, the differences in gene expression level and protein content were induced by the treatment of an individual psychotropic drug at one time point. This may not reflect the nature of many current medical regimens where acute change does not necessarily reflect chronic effects, and the concurrent use of more than two classes of psychotropic drugs is frequently seen. In the clinical setting, multiple psychotropic drugs may be used across the management time course, according to the patient’s evolving response to treatment. The interactions between these drugs and various dosages remain to be further investigated. Furthermore, at the doses used for the transcriptomics aspect of the study, none of the drugs had a significant effect on protein synthesis. This could be due to temporal effects (i.e., gene expression changes rapidly, but protein synthesis takes longer to be detectably different). It could also indicate that the measurement of gene expression is more sensitive than protein synthesis, so the effects on protein synthesis require higher doses of the drugs to be detectable. Lastly, only acute drug treatment in cell culture was performed, whilst treatment for neuropsychiatric disorders is often much longer-term (i.e., years or lifelong). It is of interest to perform drug treatment of a longer duration and investigate its effects on ribosomal gene expression and relevant pathways. Nevertheless, we have shown the effects of some psychotropic drug treatments on gene expression and overall protein production.

To summarise, we demonstrated that multiple psychotropic drugs could modulate the expression of genes involved in ribosomal function and the level of newly synthesised proteins using an in vitro human neuronal model following acute drug treatment. This approach warrants caution in interpreting the findings in the clinical context but does provide some insight into the molecular effects of these drugs in neuronal-like cells. Despite the long-standing problem of weak correlations between gene expression and protein levels, we provided the first direct evidence in the field that some psychotropic drugs generally have negative effects on ribosomal gene expression and downstream protein synthesis. Such specific effects possibly reflected the differences in mechanism of action between these drugs. Future investigations will be required to further dissect the roles of ribosomal genes and narrow down the pathways and/or functions affected by each psychotropic drug. This study provides fresh perspectives on the possibility of using protein synthesis inhibitors as new agents to treat neuropsychiatric disorders.

## 4. Materials and Methods

### 4.1. Cell Culture

A pluripotent cell line, NT2, was used as a model of human neurons. The use of this cell line and its ability to differentiate into post-mitotic neuronal NT2-N cells after treatment with retinoic acid (RA) has been described elsewhere [54,55,56,57]. In brief, NT2 cells were cultured in Dulbecco’s modified Eagle’s Medium (DMEM; Life Technologies, Melbourne, Australia) with 10% foetal bovine serum (FBS; Thermo Fisher Scientific, Melbourne, Australia) and 1% antibiotic/antimycotic solution (Life Technologies). In total, 10^−5^ M of RA was used to treat the cells for 28 days, where media change was performed every 2 to 3 days, which generated NT2-N (neuronal-like) cells. Prior to the experiments, 2 × 10^5^ cells/well of NT2-N cells were seeded onto 24-well plates coated with 10 μg/mL poly-d-lysine (Sigma-Aldrich) and 10 μg/mL laminin (Sigma-Aldrich). To enrich the culture, cells were treated with mitotic inhibitors (1 µM cytosine and 10 µM uridine; Sigma-Aldrich) in media every 2 to 3 days for a week. To validate the differentiated cells and their neuron-like phenotype, the expression of neuronal marker genes *Mash1*, *Nestin* and *GluR* was examined using polymerase chain reaction (Supplementary Figure S1).

### 4.2. Drug Treatments

NT2-N cells were treated for 24 h (*n* = 4–6) with 8 commonly prescribed psychotropic drugs purchased from Sigma-Aldrich (Sydney, Australia): amisulpride (10 µM), aripiprazole (0.1 µM), clozapine (10 µM), lamotrigine (50 µM), lithium (2.5 mM), quetiapine (50 µM), risperidone (0.1 µM) or valproate (0.5 mM). These drug doses were chosen according to previous dose–response studies in our laboratory such that no single drug dominated the overall effect on gene expression when used in combination nor affected cell viability [58]. Vehicle control cells were treated with either 0.5% Milli-Q water (controls for lithium and valproate) or 0.2% DMSO (controls for amisulpride, aripiprazole, clozapine, risperidone, lamotrigine and quetiapine).

### 4.3. Genome-Wide Gene Expression Quantification

NT2-N cells were harvested following the 24-h drug treatment using Trizol, and total RNA was extracted using RNeasy^®^ mini kits (Qiagen, Melbourne, Australia). The quality and quantity of the extracted RNA were determined using an Agilent 2100 Bioanalyzer (Agilent Technologies, Melbourne, Australia) and a NanoDrop 1000 (Thermo Fisher Scientific, Waltham, MA, USA), respectively. The preparation of RNAseq libraries for all samples from 1 µg total RNA was performed using a TruSeq RNA Sample Preparation Kit (Illumina, Victoria, Australia). To quantify genome-wide messenger RNA expression, all samples were run on an Illumina HiSeq platform (HiSeq 2500 rapid 50bpSE; 1 flow cell, 2 lanes). The raw data were processed using the Deakin Genomics Centre RNA-Seq alignment and expression quantification pipeline (available at https://github.com/m-richardson/RNASeq_pipe; last accessed on 25 May 2016) as previously described [57]. Briefly, Trimmomatic v35 was used for raw read quality filtering, and adapter trimming (ILLUMINACLIP:2:30:10:4, SLIDINGWINDOW:5:20, AVGQUAL:20 MINLEN:36) [59] and STAR v2.5 (2-pass mode) was used to align data to the reference genome (Human genome version GRCh38) [60]. For differential abundance testing, the expression was quantified at the gene level, and individual sample counts were collated into an m × n matrix. Normalisation (TMM), removal of low expressed genes (<1 cpm in *n* samples, where *n* is the number of samples in the smallest group for comparison) and differential gene expression analysis were performed using edgeR [61] in R [62] following the edgeR manual. For statistical analysis, significance was corrected for multiple testing using false discovery rate (FDR) by applying the Benjamini–Hochberg method on the *p*-values. Genes with FDR *q*-values of <0.05 were considered to be differentially expressed.

### 4.4. Gene Set Enrichment Analysis (GSEA)

GSEA was performed using the package clusterProfiler in R [63,64], where gene lists were pre-ranked based on the sign of log fold changes multiplied by the negative log10-transformed *p*-values from the differential analysis. The reference database used was the KEGG database, from which only gene sets with sizes ranging from 3 to 800 genes inclusive were considered. The resultant tables from GSEA showed enrichment scores and *p*-values calculated from 10,000 permutations, along with false discovery rate *q*-values adjusted for multiple testing.

### 4.5. Protein Synthesis Assay

Drug-treated NT2-N cells were assayed for protein synthesis using the Protein Synthesis Assay Kit from Cayman (catalogue #601100) as instructed by the manufacturer. To further assess the effects of the drugs on protein synthesis, 3 doses per drug were used to treat the cells to observe any dose-dependent effects. In brief, NT2-N cells were seeded at 20,000 cells/well in a laminin/poly-D-lysine-coated 96 well-plate and treated with psychotropic drugs of 3 different concentrations for 24 h at 37 °C, 5% CO_2_. In the last 30 min of drug treatment, cycloheximide was added to some of the wells as a negative control to inhibit protein translation. At the end of the 24h treatment, o-propargyl-puromycin dilution ([OPP] 1:400 ratio with cell culture medium) was added, followed by a 30-min incubation at 37 °C. Subsequently, cells were fixed, washed with wash buffer, stained with 5 FAM-azide staining solution (for FITC detection) and incubated at room temperature in the dark. Cells treated with only OPP were included as a positive control in which all newly translated proteins were labelled with OPP. All wells were washed with wash buffer before reading with a fluorescent plate reader (485/535 nm).

## Figures and Tables

**Table 1:** GSEA of effects of 8 psychotropic drugs on NT2-N cells.

Drug	Pathway	Set Size	NES	*p*-Value	*q*-Value
Amisulpride	Ribosome	127	−0.82	0.85	0.80
Aripiprazole	Ribosome	127	−1.54	**0.0041**	**0.033**
Clozapine	Ribosome	127	1.59	**0.0012**	**0.0048**
Lamotrigine	Ribosome	127	−1.31	**0.029**	0.15
Lithium	Ribosome	127	−1.65	**0.00072**	**0.016**
Quetiapine	Ribosome	127	0.52	1.00	0.79
Risperidone	Ribosome	127	−2.17	**0.00024**	**0.0049**
Valproate	Ribosome	127	−1.13	0.18	0.31

NES = Normalised enrichment score; *q*-value = *p*-value adjusted by the false discovery rate (FDR).

**Table 2:** Overview of the transcriptional regulation of components of the ribosome by psychotropic drugs. The direction of the arrow represents the direction of regulation (↑up- or ↓down-regulation) following drug treatment and the number of arrows represents the magnitude of *p*-value (↑ or ↓ = less than 0.05; ↑↑ or ↓↓ = less than 0.01; ↑↑↑ or ↓↓↓ = less than 0.001; NS = No significant difference).

Drug	Overall	40S Subunit	60S Subunit	Mito 28S Subunit	Mito 39S Subunit
Aripiprazole	↓↓	↓	↓↓	NS	NS
Clozapine	↑↑	↑↑	↑↑↑	NS	NS
Lithium	↓↓↓	↓↓↓	↓↓↓	NS	NS
Risperidone	↓↓↓	↓↓	↓↓↓	↓↓↓	↓↓

## Data Availability

Data available from authors upon reasonable request.

## Supplementary Materials

The following supporting information can be downloaded at: https://www.mdpi.com/article/10.3390/ijms23137180/s1.

## References

[1] Porokhovnik L.N., Passekov V.P., Gorbachevskaya N.L., Sorokin A.B., Veiko N.N., Lyapunova N.A. (2015). Active ribosomal genes, translational homeostasis and oxidative stress in the pathogenesis of schizophrenia and autism. Psychiatr. Genet..

[2] Chestkov I.V., Jestkova E.M., Ershova E.S., Golimbet V.E., Lezheiko T.V., Kolesina N.Y., Porokhovnik L.N., Lyapunova N.A., Izhevskaya V.L., Kutsev S.I. (2018). Abundance of ribosomal RNA gene copies in the genomes of schizophrenia patients. Schizophr. Res..

[3] Hori H., Nakamura S., Yoshida F., Teraishi T., Sasayama D., Ota M., Hattori K., Kim Y., Higuchi T., Kunugi H. (2018). Integrated profiling of phenotype and blood transcriptome for stress vulnerability and depression. J. Psychiatr. Res..

[4] Tian T., Wei Z., Chang X., Liu Y., Gur R.E., Sleiman P.M.A., Hakonarson H. (2018). The Long Noncoding RNA Landscape in Amygdala Tissues from Schizophrenia Patients. EBioMedicine.

[5] Han K., Holder J.L., Schaaf C.P., Lu H., Chen H., Kang H., Tang J., Wu Z., Hao S., Cheung S.W. (2013). SHANK3 overexpression causes manic-like behaviour with unique pharmacogenetic properties. Nature.

[6] Gauthier J., Champagne N., Lafrenière R.G., Xiong L., Spiegelman D., Brustein E., Lapointe M., Peng H., Côté M., Noreau A. (2010). De novo mutations in the gene encoding the synaptic scaffolding protein SHANK3 in patients ascertained for schizophrenia. Proc. Natl. Acad. Sci. USA.

[7] Lee Y., Zhang Y., Kim S., Han K. (2018). Excitatory and inhibitory synaptic dysfunction in mania: An emerging hypothesis from animal model studies. Exp. Mol. Med..

[8] Jin C., Kang H., Ryu J.R., Kim S., Zhang Y., Lee Y., Kim Y., Han K. (2018). Integrative Brain Transcriptome Analysis Reveals Region-Specific and Broad Molecular Changes in Shank3-Overexpressing Mice. Front. Mol. Neurosci..

[9] Wilson D.N., Doudna Cate J.H. (2012). The structure and function of the eukaryotic ribosome. Cold Spring Harb. Perspect. Biol..

[10] Lafontaine D.L., Tollervey D. (2001). The function and synthesis of ribosomes. Nat. Rev. Mol. Cell Biol..

[11] Clay H.B., Sillivan S., Konradi C. (2011). Mitochondrial dysfunction and pathology in bipolar disorder and schizophrenia. Int. J. Dev. Neurosci..

[12] Tobe E.H. (2013). Mitochondrial dysfunction, oxidative stress, and major depressive disorder. Neuropsychiatr. Dis. Treat..

[13] Greber B.J., Ban N. (2016). Structure and function of the mitochondrial ribosome. Annu. Rev. Biochem..

[14] Laguesse S., Ron D. (2020). Protein Translation and Psychiatric Disorders. Neuroscientist.

[15] Forrest M.P., Parnell E., Penzes P. (2018). Dendritic structural plasticity and neuropsychiatric disease. Nat. Rev. Neurosci..

[16] Pfaffenseller B., Wollenhaupt-Aguiar B., Fries G.R., Colpo G.D., Burque R.K., Bristot G., Ferrari P., Ceresér K.M., Rosa A.R., Klamt F. (2014). Impaired endoplasmic reticulum stress response in bipolar disorder: Cellular evidence of illness progression. Int. J. Neuropsychopharmacol..

[17] Karyo R., Eskira Y., Pinhasov A., Belmaker R., Agam G., Eldar-Finkelman H. (2010). Identification of eukaryotic elongation factor-2 as a novel cellular target of lithium and glycogen synthase kinase-3. Mol. Cell. Neurosci..

[18] Fusco C.M., Desch K., Dörrbaum A.R., Wang M., Staab A., Chan I.C., Vail E., Villeri V., Langer J.D., Schuman E.M. (2021). Neuronal ribosomes exhibit dynamic and context-dependent exchange of ribosomal proteins. Nat. Commun..

[19] English J.A., Fan Y., Föcking M., Lopez L.M., Hryniewiecka M., Wynne K., Dicker P., Matigian N., Cagney G., Mackay-Sim A. (2015). Reduced protein synthesis in schizophrenia patient-derived olfactory cells. Transl. Psychiatry.

[20] Heaney C.F., Raab-Graham K.F. (2018). Dysregulated Protein Synthesis in Major Depressive Disorder. The Oxford Handbook of Neuronal Protein Synthesis.

[21] Arion D., Huo Z., Enwright J.F., Corradi J.P., Tseng G., Lewis D.A. (2017). Transcriptome alterations in prefrontal pyramidal cells distinguish schizophrenia from bipolar and major depressive disorders. Biol. Psychiatry.

[22] Al Eissa M.M., Sharp S.I., O’Brien N.L., Fiorentino A., Bass N.J., Curtis D., McQuillin A. (2019). Genetic association and functional characterization of *MCPH1* gene variation in bipolar disorder and schizophrenia. Am. J. Med. Genet. B Neuropsychiatr. Genet..

[23] Krull F., Akkouh I., Hughes T., Bettella F., Athanasiu L., Smeland O.B., O’Connell K.S., Brattbakk H.R., Steen V.M., Steen N.E. (2022). Dose-dependent transcriptional effects of lithium and adverse effect burden in a psychiatric cohort. Prog. Neuro-Psychopharmacol. Biol. Psychiatry.

[24] Akkouh I.A., Skrede S., Holmgren A., Ersland K.M., Hansson L., Bahrami S., Andreassen O.A., Steen V.M., Djurovic S., Hughes T. (2020). Exploring lithium’s transcriptional mechanisms of action in bipolar disorder: A multi-step study. Neuropsychopharmacology.

[25] Yelick P.C., Trainor P.A. (2015). Ribosomopathies: Global process, tissue specific defects. Rare Dis..

[26] Paolini N.A., Attwood M., Sondalle S.B., Vieira C.M.D.S., van Adrichem A.M., di Summa F.M., O’Donohue M.-F., Gleizes P.-E., Rachuri S., Briggs J.W. (2017). A Ribosomopathy Reveals Decoding Defective Ribosomes Driving Human Dysmorphism. Am. J. Hum. Genet..

[27] Chakraborty A., Uechi T., Higa S., Torihara H., Kenmochi N. (2009). Loss of ribosomal protein L11 affects zebrafish embryonic development through a p53-dependent apoptotic response. PLoS ONE.

[28] Wang M., Hu Y., Stearns M.E. (2009). RPS2: A novel therapeutic target in prostate cancer. J. Exp. Clin. Cancer Res..

[29] Costantini A., Alm J.J., Tonelli F., Valta H., Huber C., Tran A.N., Daponte V., Kirova N., Kwon Y.U., Bae J.Y. (2021). Novel RPL13 Variants and Variable Clinical Expressivity in a Human Ribosomopathy With Spondyloepimetaphyseal Dysplasia. J. Bone Miner. Res..

[30] Guan J., Han S., Wu J., Zhang Y., Bai M., Abdullah S.W., Sun S., Guo H. (2021). Ribosomal Protein L13 Participates in Innate Immune Response Induced by Foot-and-Mouth Disease Virus. Front. Immunol..

[31] Wei X., Zhang Y., Fu Z., Zhang L. (2013). The association between polymorphisms in the MRPL4 and TNF-α genes and susceptibility to allergic rhinitis. PLoS ONE.

[32] Andiappan A.K., Wang D.Y., Anantharaman R., Parate P.N., Suri B.K., Low H.Q., Li Y., Zhao W., Castagnoli P., Liu J. (2011). Genome-wide association study for atopy and allergic rhinitis in a Singapore Chinese population. PLoS ONE.

[33] Gardeitchik T., Mohamed M., Ruzzenente B., Karall D., Guerrero-Castillo S., Dalloyaux D., van den Brand M., van Kraaij S., van Asbeck E., Assouline Z. (2018). Bi-allelic Mutations in the Mitochondrial Ribosomal Protein MRPS2 Cause Sensorineural Hearing Loss, Hypoglycemia, and Multiple OXPHOS Complex Deficiencies. Am. J. Hum. Genet..

[34] Liu C., Zhou W., Liu Q., Peng Z. (2022). Hypoglycemia with lactic acidosis caused by a new MRPS2 gene mutation in a Chinese girl: A case report. BMC Endocr. Disord..

[35] Serre V., Rozanska A., Beinat M., Chretien D., Boddaert N., Munnich A., Rötig A., Chrzanowska-Lightowlers Z.M. (2013). Mutations in mitochondrial ribosomal protein MRPL12 leads to growth retardation, neurological deterioration and mitochondrial translation deficiency. Biochim. Biophys. Acta (BBA)-Mol. Basis Dis..

[36] Khokhar J.Y., Henricks A.M., Sullivan E.D.K., Green A.I. (2018). Unique Effects of Clozapine: A Pharmacological Perspective. Adv. Pharmacol..

[37] Greenbaum D., Colangelo C., Williams K., Gerstein M. (2003). Comparing protein abundance and mRNA expression levels on a genomic scale. Genome Biol..

[38] Nie L., Wu G., Culley D.E., Scholten J.C., Zhang W. (2007). Integrative analysis of transcriptomic and proteomic data: Challenges, solutions and applications. Crit. Rev. Biotechnol..

[39] Gry M., Rimini R., Strömberg S., Asplund A., Pontén F., Uhlén M., Nilsson P. (2009). Correlations between RNA and protein expression profiles in 23 human cell lines. BMC Genom..

[40] Waters K.M., Pounds J.G., Thrall B.D. (2006). Data merging for integrated microarray and proteomic analysis. Brief. Funct. Genom. Proteomic.

[41] Edfors F., Danielsson F., Hallström B.M., Käll L., Lundberg E., Pontén F., Forsström B., Uhlén M. (2016). Gene-specific correlation of RNA and protein levels in human cells and tissues. Mol. Syst. Biol..

[42] Prabhakar U., Conway T.M., Murdock P., Mooney J.L., Clark S., Hedge P., Bond B.C., Jazwinska E.C., Barnes M.R., Tobin F. (2005). Correlation of protein and gene expression profiles of inflammatory proteins after endotoxin challenge in human subjects. DNA Cell Biol..

[43] Baig M.R., Navaira E., Escamilla M.A., Raventos H., Walss-Bass C. (2010). Clozapine treatment causes oxidation of proteins involved in energy metabolism in lymphoblastoid cells: A possible mechanism for antipsychotic-induced metabolic alterations. J. Psychiatr. Pract..

[44] Šunderić M., Vasović T., Milčić M., Miljević Č., Nedić O., Nikolić M.R., Gligorijević N. (2021). Antipsychotic clozapine binding to alpha-2-macroglobulin protects interacting partners against oxidation and preserves the anti-proteinase activity of the protein. Int. J. Biol. Macromol..

[45] Bader N., Grune T. (2006). Protein oxidation and proteolysis. Biol. Chem..

[46] Mavrikaki M., Schintu N., Kastellakis A., Nomikos G.G., Svenningsson P., Panagis G. (2014). Effects of lithium and aripiprazole on brain stimulation reward and neuroplasticity markers in the limbic forebrain. Eur. Neuropsychopharmacol..

[47] Park S.Y., Shin H.K., Lee W.S., Bae S.S., Kim K., Hong K.W., Kim C.D. (2017). Neuroprotection by aripiprazole against β-amyloid-induced toxicity by P-CK2α activation via inhibition of GSK-3β. Oncotarget.

[48] Shin S., Wolgamott L., Yu Y., Blenis J., Yoon S.O. (2011). Glycogen synthase kinase (GSK)-3 promotes p70 ribosomal protein S6 kinase (p70S6K) activity and cell proliferation. Proc. Natl. Acad. Sci. USA.

[49] Brandão-Teles C., de Almeida V., Cassoli J.S., Martins-de-Souza D. (2019). Biochemical Pathways Triggered by Antipsychotics in Human Oligodendrocytes: Potential of Discovering New Treatment Targets. Front. Pharmacol..

[50] Wang X., Proud C.G. (2006). The mTOR pathway in the control of protein synthesis. Physiology.

[51] Tang S.J., Reis G., Kang H., Gingras A.-C., Sonenberg N., Schuman E.M. (2002). A rapamycin-sensitive signaling pathway contributes to long-term synaptic plasticity in the hippocampus. Proc. Natl. Acad. Sci. USA.

[52] Ma T., Hoeffer C.A., Capetillo-Zarate E., Yu F., Wong H., Lin M.T., Tampellini D., Klann E., Blitzer R.D., Gouras G.K. (2010). Dysregulation of the mTOR pathway mediates impairment of synaptic plasticity in a mouse model of Alzheimer’s disease. PLoS ONE.

[53] Zheng L., Yang L., Zhao X., Long N., Li P., Wang Y. (2019). Effect of risperidone on proliferation and apoptosis of MC3T3-E1 cells. Braz. J. Med. Biol. Res..

[54] Pleasure S.J., Page C., Lee V. (1992). Pure, postmitotic, polarized human neurons derived from NTera 2 cells provide a system for expressing exogenous proteins in terminally differentiated neurons. J. Neurosci..

[55] Megiorni F., Mora B., Indovina P., Mazzilli M.C. (2005). Expression of neuronal markers during NTera2/cloneD1 differentiation by cell aggregation method. Neurosci. Lett..

[56] Bortolasci C.C., Spolding B., Callaly E., Martin S., Panizzutti B., Kidnapillai S., Connor T., Hasebe K., Mohebbi M., Dean O.M. (2018). Mechanisms underpinning the polypharmacy effects of medications in psychiatry. Int. J. Neuropsychopharmacol..

[57] Bortolasci C.C., Spolding B., Kidnapillai S., Connor T., Truong T.T., Liu Z.S., Panizzutti B., Richardson M.F., Gray L., Berk M. (2020). Transcriptional Effects of Psychoactive Drugs on Genes Involved in Neurogenesis. Int. J. Mol. Sci..

[58] Panizzutti B., Bortolasci C.C., Spolding B., Kidnapillai S., Connor T., Richardson M.F., Truong T.T.T., Liu Z.S.J., Morris G., Gray L. (2021). Transcriptional Modulation of the Hippo Signaling Pathway by Drugs Used to Treat Bipolar Disorder and Schizophrenia. Int. J. Mol. Sci..

[59] Bolger A.M., Lohse M., Usadel B. (2014). Trimmomatic: A flexible trimmer for Illumina sequence data. Bioinformatics.

[60] Dobin A., Davis C.A., Schlesinger F., Drenkow J., Zaleski C., Jha S., Batut P., Chaisson M., Gingeras T.R. (2013). STAR: Ultrafast universal RNA-seq aligner. Bioinformatics.

[61] Robinson M.D., McCarthy D.J., Smyth G.K. (2010). edgeR: A Bioconductor package for differential expression analysis of digital gene expression data. Bioinformatics.

[62] R Core Team (2013). R: A Language and Environment for Statistical Computing.

[63] Yu G., Wang L.-G., Han Y., He Q.-Y. (2012). clusterProfiler: An R package for comparing biological themes among gene clusters. Omics A J. Integr. Biol..

[64] Subramanian A., Tamayo P., Mootha V.K., Mukherjee S., Ebert B.L., Gillette M.A., Paulovich A., Pomeroy S.L., Golub T.R., Lander E.S. (2005). Gene set enrichment analysis: A knowledge-based approach for interpreting genome-wide expression profiles. Proc. Natl. Acad. Sci. USA.

