# Supplementary material for: Effects of Psychotropic Drugs on Ribosomal Genes and Protein Synthesis
Source: Int J Mol Sci. 2022 Jun 28;23(13):7180. doi: 10.3390/ijms23137180 (PMC9266764; doi:10.3390/ijms23137180)
Supplement: Supplementary file 1 [file ijms-23-07180-s001.zip › ijms-1771737-supplementary.pdf]

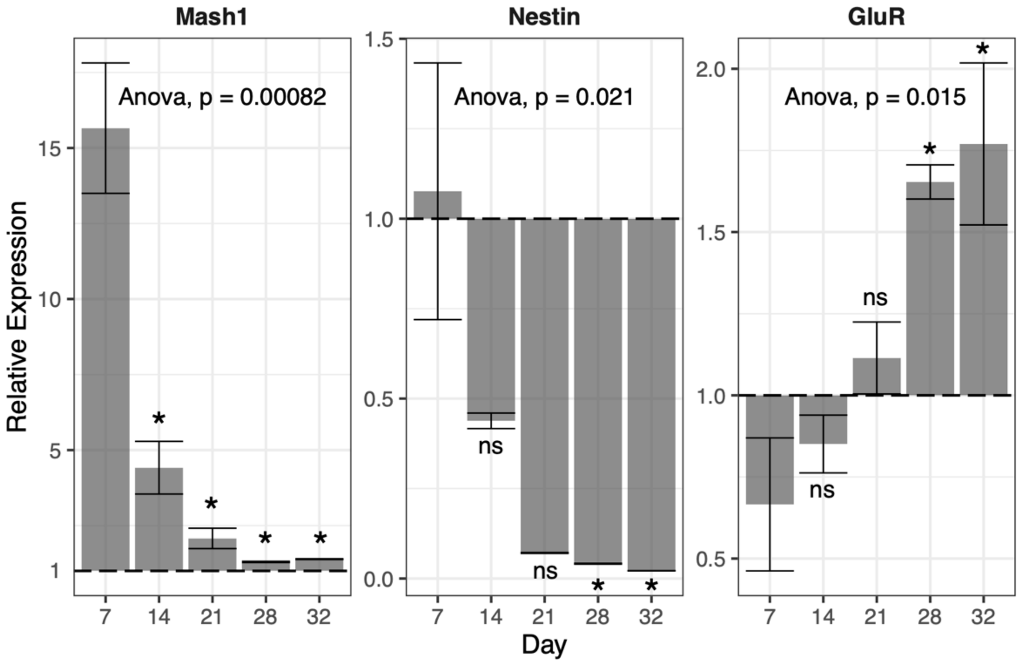


Figure S1: The relative expression level of three neuronal marker genes in NT2-N cell culture. The panel included a pro-neural transcriptional factor marker (*Mash1*), a neuronal stem and progenitor cell marker (*Nestin*) and a NT2 neuronal differentiation marker (*GluR*). The expression levels per gene were normalised by the mean of Day 0 data. ANOVA and non-parametric t test were performed to examine the difference in the mean expression level between days (day 7 was used as refeference). ns represents $p > 0.05$ and * $p \leq 0.05$.
